# Sulforaphane, a natural component of broccoli, inhibits vestibular schwannoma growth *in vitro* and *in vivo*

**DOI:** 10.1038/srep36215

**Published:** 2016-11-02

**Authors:** Bo Gyung Kim, Takeshi Fujita, Konstantina M. Stankovic, D. Bradley Welling, In Seok Moon, Jae Young Choi, Jieun Yun, Jong Soon Kang, Jong Dae Lee

**Affiliations:** 1 Department of Otorhinolaryngology-Head and Neck Surgery, Soonchunhyang University College of Medicine, Bucheon, Korea; 2 Department of Otolaryngology, Kindai University Faculty of Medicine, Osaka, Japan; 3 Eaton Peabody Laboratories, Department of Otolaryngology, Massachusetts Eye and Ear Infirmary, Boston, MA, USA; 4 Department of Otology and Laryngology, Harvard Medical School, Boston, MA, USA; 5 Department of Otorhinolaryngology, Yonsei University College of Medicine, Seoul, Korea; 6 Bioevaluation Center, Korea Research Institute of Bioscience and Biotechnology, Cheongju, Korea

## Abstract

Vestibular schwannoma (VS) is an intracranial tumor that causes significant morbidity, including hearing loss, tinnitus, dizziness, and possibly even death from brainstem compression. However, FDA-approved pharmacologic treatments for VS do not exist. Sulforaphane (SFN) is a naturally occurring isothiocyanate found in cruciferous vegetables, such as broccoli, with potent chemoprotective effects in several cell types. Our objective was to determine whether SFN is effective against VS *in vitro* and *in vivo*. Human primary VS cells, HEI-193 schwannoma cells, and SC4 Nf2^−/−^ Schwann cells were used to investigate the inhibitory effects of SFN *in vitro*. Cell proliferation was assessed by bromodeoxyuridine (BrdU) incorporation, and cell viability and metabolic activity was calculated by MTT assay. Apoptosis was measured by flow cytometry, terminal deoxynucleotidyl transferase-mediated dUTP nick end labeling (TUNEL) staining, and Western blot for cleaved caspases. A mouse model with a murine schwannoma allograft was also used to examine the antitumor activity of SFN. SFN exhibited significant antiproliferative activity in schwannoma cells *in vitro*, via the inhibition of HDAC activity and the activation of ERK. SFN treatment induced apoptosis and cell cycle arrest at the G2/M phase. SFN also significantly inhibited schwannoma growth *in vivo*. Our preclinical studies motivate a future prospective clinical study of SFN for the treatment of VS.

Vestibular schwannoma (VS) is a non-malignant tumor originating from Schwann cells of the vestibular nerve. VSs occur either sporadically and unilaterally, or bilaterally as hallmark tumors of neurofibromatosis type 2 (NF2). VSs result in significant morbidity, including hearing loss, tinnitus and dizziness. As these tumors grow, they can cause facial paralysis and other cranial neuropathies, as well as death from brainstem compression. Although microsurgery and stereotactic radiation remain the mainstay of treatment for growing VSs, observation is an acceptable option for newly diagnosed and non-growing tumors. At present, since the majority of VSs are diagnosed early, observation is the most common initial choice^1^. Such a choice is justified because both microsurgery and stereotactic radiation therapy carry significant risks, including the risk of irreversible ipsilateral hearing loss. Despite the slow progression of the disease, no pharmacologic treatments have been approved for VS, highlighting the need to develop effective and well-tolerated pharmaceutical therapies.

Several clinical trials for NF2-associated VSs are currently underway, targeting key pathways important for disease pathogenesis^2^,^3^. However, these trials have relied on expensive chemotherapeutic agents with substantial adverse effects, including cardiovascular toxicity^4^, so widespread use of these drugs to treat non-malignant tumors is unlikely. The unmet medical need to identify safe agents with minimal adverse effects to treat VS has motivated our study of dietary agents with potential anti-tumoral activities. Dietary agents found in fruit and vegetables have been shown to interfere with multiple cellular signaling pathways important in cancer pathogenesis, and several of these agents are capable of not only preventing but even treating some cancers^5^. We focus on sulforaphane, a dietary agent extracted from cruciferous vegetables, and its potential to inhibit VS growth.

Sulforaphane (SFN) is an isothiocyanate found in cruciferous vegetables, such as broccoli. SFN has been shown to exhibit chemoprotective effects for various tumors, including those of the prostate, lung, breast and colon, *in vitro* and *in vivo*^6^,^7^,^8^. These studies have shown that SFN significantly affects cell survival and leads to apoptosis through a variety of molecular mechanisms, including the potent inhibition of histone deacetylase (HDAC)^9^,^10^,^11^. As there have been no reports examining the effect of SFN on vestibular schwannoma, here we characterize the ilnhibitory effects of SFN on VS cells *in vitro* using primary human VS cells and VS-derived cell lines, and *in vivo* using a mouse model with a murine schwannoma allograft.

## Results

### Sulforaphane inhibits the proliferation of primary human vestibular schwannoma cells and an NF2-derived cell line *in vitro*

**Figure 1:**
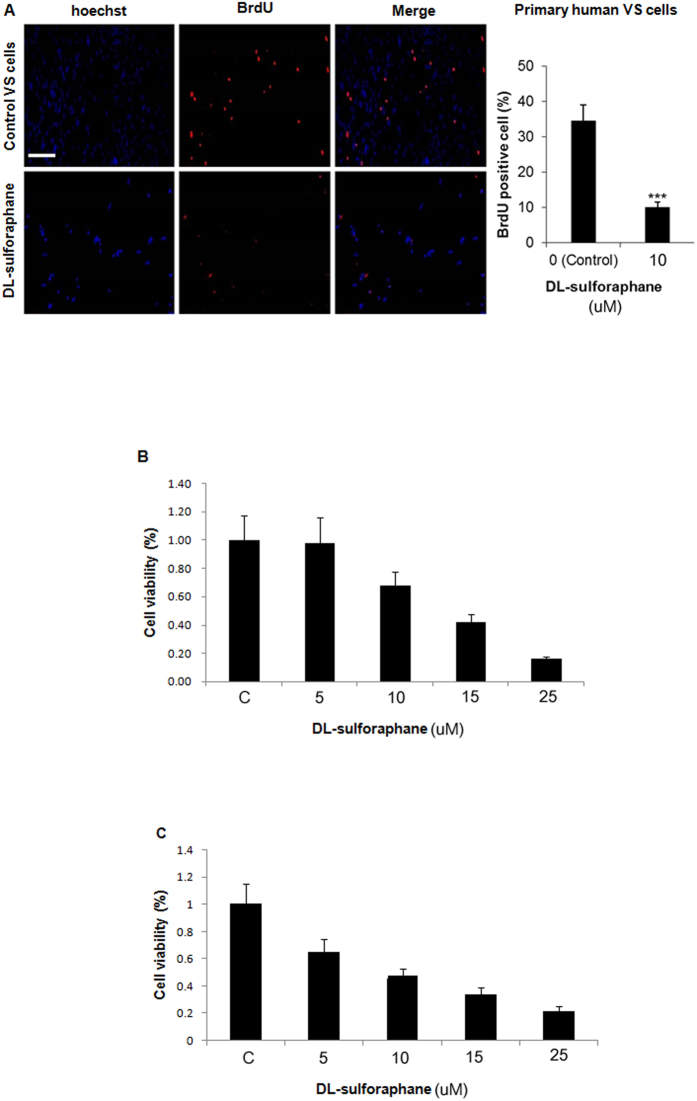
Sulforaphane inhibits proliferation of primary human vestibular schwannoma (VS) cells in a dose-dependent manner. (**A**) Proliferation of primary human VS cells derived from a combination of 3 different VSs, as measured using bromodeoxyuridine (BrdU) incorporation (red), was inhibited by application of sulforaphane (10 μM) for 48 h. BrdU incorporation was calculated as the percentage of BrdU-labeled nuclei relative to total nuclei stained with Hoechst33342. Scale bar = 100 μM (same scale bar applies to all). Bar graph depicts mean ± standard error of mean (SEM, n = 9). ***p < 0.001. **(B)** Cell viability (measured with MTT assay) of human HEI-193 cells and **(C)** mouse SC4 Nf2^−/−^ Schwann cells incubated with 5–25 μM concentrations of sulforaphane for 48 h. Bar graphs represent mean ± SEM (n = 6).

After 48 hours of incubation with SFN (10 μM), BrdU was used to assess proliferation in cells grown from three different primary human VSs, compared to untreated controls (Fig. 1A). SFN substantially and significantly (p < 0.001) reduced BrdU incorporation by primary VS cells, consistent with an anti-proliferative effect. This inhibitory effect of SFN was confirmed using two different cell lines and a different assay. Specifically, human HEI-193 cells (Fig. 1B) and mouse SC4 cells (Fig. 1C) were treated with 5, 10, 15 or 25 μM of SFN or vehicle control (DMSO) for 48 hours, and cell viability was assessed using an MTT assay. SFN inhibited cell viability in a dose-dependent manner.

### Sulforaphane induces apoptosis in human vestibular schwannoma cells

**Figure 2:**
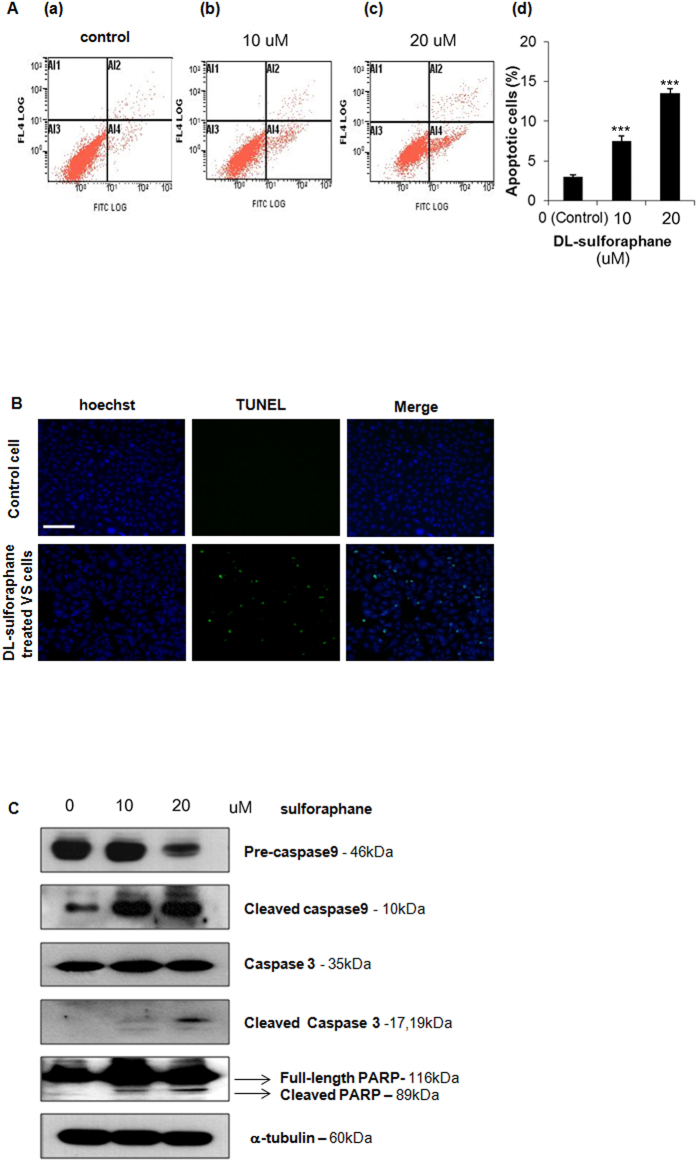
Effect of sulforaphane on apoptosis in HEI-193 cells. (**A**) Cell death quantified by Annexin V-PI staining after application of 0 (a), 10 (b), or 20 (c) μM SFN for 48 h. Apoptotic cells appear in the right upper and right lower quadrant. Bar graph (d) depicts mean ± standard error of mean (SEM, n = 6). ***p < 0.001. (**B**) Apoptosis detected by terminal deoxynucleotidyl transferase-mediated dUTP nick end labeling (TUNEL). Scale bar = 100 μm (applies to all). (**C**) Expression of cleaved caspase-3, -9, and poly-ADP ribose polymerase (full-length and cleaved bands), detected by Western blot.

Human HEI-193 cells were used to quantify the effect of SFN on apoptosis using annexin V-propidium iodide (PI) staining and TUNEL staining. In annexin V-PI staining assays, the relative strength of fluorescein isothiocyanate (FITC) and PI (FL4) fluorescence reflects cell health and is depicted in 4 quadrants (Fig. 2A): 1) dead cells exhibit weak FITC fluorescence and strong PI fluorescence (left upper quadrant, known as quadrant Al1); 2) late apoptotic cells exhibit strong FITC and strong PI fluorescence (right upper quadrant, known as quadrant Al2); 3) healthy cells exhibit low levels of FITC and PI fluorescence (left lower quadrant, known as quadrant Al3); 4) early apoptotic cells exhibit strong FITC and weak PI fluorescence (right lower quadrant, known as quadrant A14). Compared to HEI-193 cells treated with vehicle control (Fig. 2A(a)), HEI-193 cells treated with 10 μM SFN (Fig. 2A(b) and 20 μM SFN (Fig. 2A(c)) had significantly higher proportions of apoptotic cells (Al2 + Al4), as summarized in Fig. 2A(d).

Sulforaphane-induced apoptosis in HEI-193 cells was demonstrated further using TUNEL staining *in situ*, which detects DNA fragmentation. A significant increase in both DNA fragmentation and perinuclear apoptotic body formation were seen in HEI-193 cells treated with 10 μM SFN compared to control cells treated with vehicle only (Fig. 2B).

Apoptotic assays based on staining characteristics (Fig. 2A) and DNA fragmentation (Fig. 2B) were complemented by measuring proteins involved in apoptosis by Western blot (Fig. 2C). Specifically, we measured expression of caspase-9, caspase-3, and caspase-3-mediated poly (ADP-ribose) polymerase (PARP), as well as the cleaved forms of these proteins. In HEI-193 cells treated with 20 μM SFN for 48 h relative to controls, the expression of pre-caspase-9 (precursor form) significantly decreased, while the expression of cleaved caspase-9 increased. Cleavage of caspase-3 was used as a marker of cells undergoing apoptosis, while the observed cleavage of PARP was consistent with caspase-mediated apoptosis. Taken together, these data confirm SFN-induced apoptosis in HEI-193 cells via induction of caspase activity.

### Sulforaphane induces cell cycle arrest

**Figure 3:**
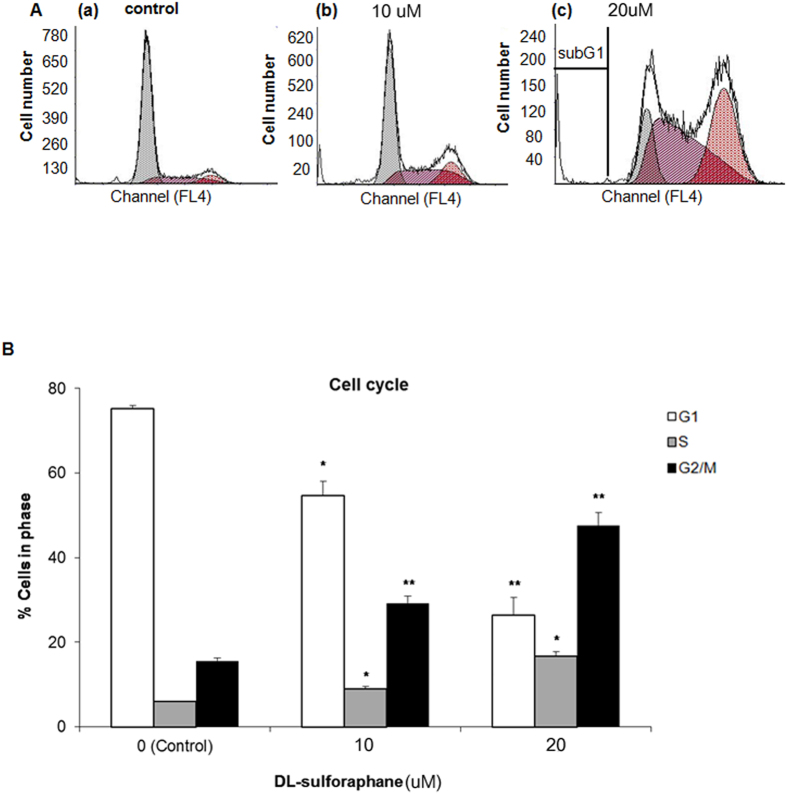
Cell cycle in HEI-193 cells is arrested by sulforaphane treatment. (**A**) The distribution of cell cycle, as determined by propodium iodide labeling, after application of 0 (a), 10 (b), or 20 (c) μM SFN for 48 h. DNA content was analyzed by flow cytometry. (**B**) Proportion of cells per phase of cell cycle. Data are depicted as mean ± standard error of mean (SEM, n = 3). *p < 0.05; **p < 0.01.

Because arresting the cell cycle can have a therapeutic effect for various neoplasms^10^,^12^, we studied whether sulforaphane arrested the cell cycle of HEI-193 cells (Fig. 3). SFN significantly arrested schwannoma cells in the G2/M phase of the cell cycle in a concentration-dependent manner (Fig. 3A). When comparing 0 versus 20 μM SFN, the proportion of HEI-193 cells in G2/M phase increased from 15.57 ± 0.79 to 47.68 ± 2.96% (p < 0.01), implying G2/M arrest (Fig. 3B).

### Sulforaphane inhibits histone deacetylase activity and increases ERK in schwannoma cells

**Figure 4:**
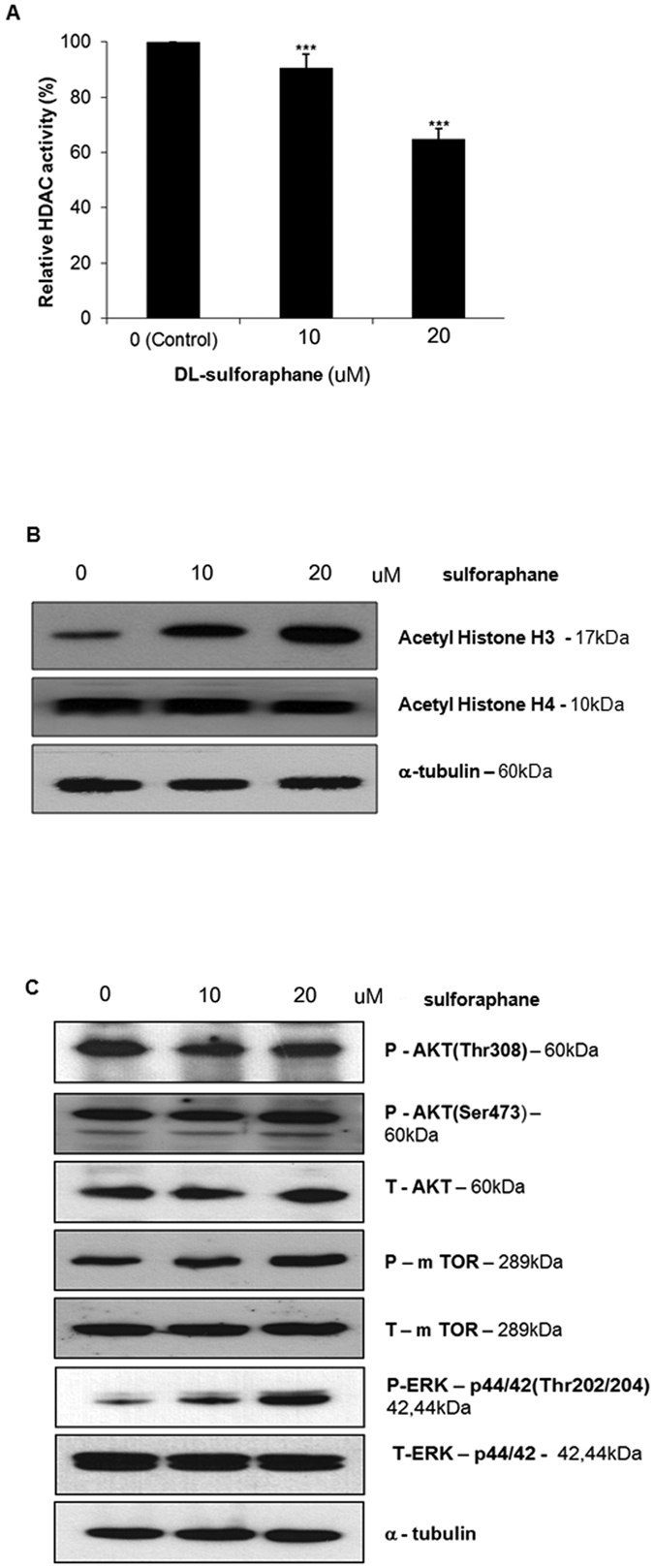
Sulforaphane inhibits histone deacetylase (HDAC) and activates ERK 1/2 kinases in human HEI-193 cells. (**A**) SFN inhibits histone deacetylase activity in HEI-193 cells, as measured with a fluorometric kit. Boxes represent as mean ± standard error of mean (SEM, n = 6). ***p < 0.001. **(B)** Acetylation of histones H3 and H4, as assessed by Western blot, following application of 0, 10, or 20 μM SFN for 48 h. (**C**) Activation of ERK, as assessed by western blot, following application of 0, 10, or 20 μM SFN for 48 h; α-tubulin was used as the loading control. P: phosphor forms. T: total forms.

Because sulforphane is a potent inhibitor of histone deacetylase (HDAC) in several malignancies^7^, we studied whether it had a similar effect on non-malignant schwannoma cells. HDAC activity was inhibited by SFN in a dose-dependent manner in HEI-193 cells (Fig. 4A). Moreover, expression of acetyl histone H3 was significantly increased in a dose-dependent manner, as assessed by Western blot (Fig. 4B). Regulation of the AKT/mTOR signaling kinases and extracellular signal-regulated kinase (ERK) has previously been observed in response to SFN treatment in breast cancer, prostate cancer, and hepatoma^6^,^13^,^14^. SFN induced activation of ERK in HEI-193 cells but had no effect on AKT/mTOR (Fig. 4C).

### Sulforaphane inhibits schwannoma growth *in vivo*

**Figure 5:**
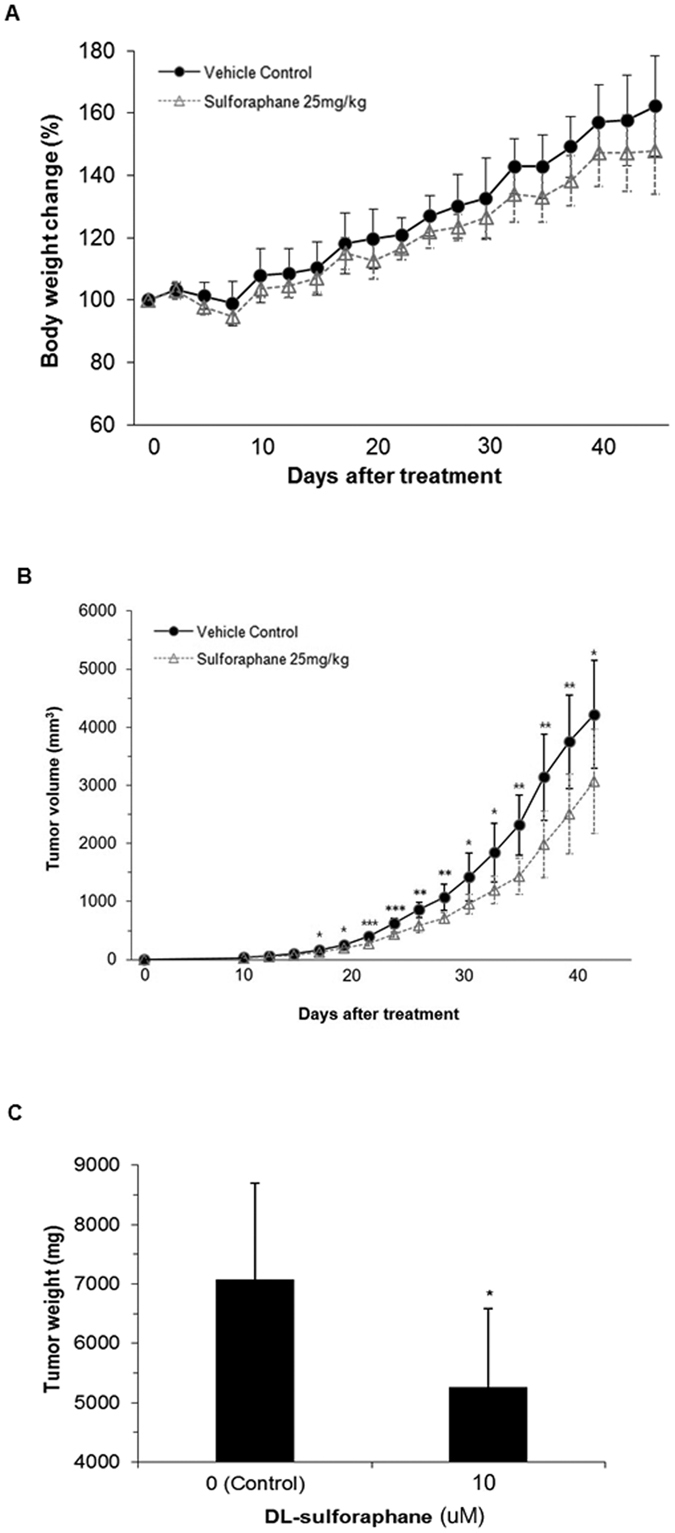
Sulforaphane decreases tumor volume *in vivo*. (**A**) Body weight, (**B**) tumor volume, and (**C**) final tumor weight in mice receiving sulforaphane (25 mg/kg, n = 8) or vehicle control (n = 8). Data points and bar graphs depict mean ± S.D; *p < 0.05, **p < 0.05, ***p < 0.001 compared with vehicle control.

To examine the effect of SFN on schwannomas *in vivo*, mouse SC4 Nf2^−/−^ Schwann cells were allografted to nude mice. Mice were treated with SFN or vehicle control via intraperitoneal administration five times per week for 6 weeks, after which we compared tumor volumes between groups. No statistically significant change in mean body weight between SFN-treated (n = 8 mice) and control animals (vehicle only, n = 8 mice) was observed over the course of the study, suggesting that SFN was not toxic (Fig. 5A). SFN-treated mice (25 mg/kg) showed a statistically significant inhibition of tumor growth at all time points from 16 days after treatment on. At the completion of treatment a 27.2% reduction in tumor volume (p < 0.05) was observed compared with the vehicle control group (Fig. 5B). Tumor weight was also significantly lower at the completion of treatment, with the SFN group exhibiting a 25.8% reduction in mean tumor weight relative to the vehicle control group (p < 0.05; Fig. 5C).

## Discussion

We have shown, for the first time, that a dietary extract, sulforaphane, is therapeutic against human vestibular schwannoma cells *in vitro* and mouse schwannoma cells *in vivo*. Significant insights into the pathways underlying VS pathogenesis have led to a variety of new therapeutic targets for intervention in schwannoma. Targeted molecular therapies for VS using EGFR/ErBb2 (lapatinib), mTOR (everolimus), and VEGF inhibitor (bevacizumab) have been examined in patients with NF2^15^,^16^,^17^. However, the cost and toxicity associated with these treatments may be inappropriate for non-malignant tumors, such as VS. Alternative treatments, based on safe dietary supplements capable of slowing the progression of VS, may represent a more practical approach for many patients. For this reason, we have reported three alternative natural products capable of inhibiting the growth of HEI-193 cells^18^,^19^,^20^. Furthermore, Spear *et al*. reported that cucurbitacin D and goyazensolide exhibit anti-proliferative activity in *NF2*-deficient schwannoma and meningioma cells, though neither has been tested in primary VS cells or in *in vivo* models^21^.

Recent studies have identified a variety of micronutrients present in fruits and vegetables, including curcumin, resveratrol, genistein, diallyl sulfide, S-allyl cysteine, allicin, lycopene, capsaicin, diosgenin, 6-gingerol, ellagic acid, ursolic acid, silymarin, anethol, catechins, eugenol, limonene, beta carotene, and SFN, which appear to limit tumor growth, suggesting a mechanism by which dietary constituents may exert anti-carcinogenic activity^5^,^22^. Here, we sought to identify compounds with inhibitory effects on schwannoma cells at doses consistent with their natural concentrations. We found that SFN had inhibitory effects on primary human vestibular schwannoma cells and a human NF2-derived VS cell line *in vitro*, as well as on an *in vivo* allograft model of VS.

HDAC inhibitors are emerging as a new class of antitumor drugs, due to their ability to promote differentiation, induce cell cycle arrest, and trigger apoptosis^23^,^24^. HDAC inhibitors have been shown to inhibit multiple intracranial tumors both *in vitro* and *in vivo* through a variety of mechanisms^25^,^26^. Among these, a novel HDAC inhibitor, AR42, has shown inhibitory activity against NF2-associated tumors via the induction of apoptosis^27^,^28^. SFN has also been shown to inhibit HDAC activity in several human cancers, as evidenced by acetylation of histones H3 and H4, two common markers for successful HDAC inhibition^9^,^10^. Our data presented here show that SFN treatment results in successful upregulation of acetylated histone H3 in VS cells, increased apoptosis, and cell cycle arrest, consistent with HDAC inhibition.

Given the complexity of the signaling pathways downstream of merlin/NF2, a number of potential therapeutic targets have been proposed^3^. The MAPK/ERK and AKT-mTOR pathways are two of the major downstream signaling pathways in schwannomas^29^. Several studies have shown that HDAC inhibitors may suppress these pathways in some tumors^30^,^31^; specifically, AR42, a novel HDAC inhibitor, has been shown to reduce VS proliferation through AKT signaling^27^. Additionally, it has been reported that SFN inhibits cell proliferation via activation of ERK in prostate tumors and hepatomas^6^,^7^. Our data support the conclusion that SFN induces activation of ERK, without affecting AKT-mTOR in VS cells.

Although dietary agents can be used in their natural forms to inhibit tumor progression, large doses are often necessary to achieve significant therapeutic outcomes. While these agents are pharmacologically safe in most situations, one of the main concerns is the lack of bioavailability. However, the bioavailability of these compounds should not be evaluated in the same manner as synthetic compounds, as many of these agents exhibit biological responses at serum concentrations that are otherwise undetectable *in vitro*^5^. It is known that dietary intake of sulforaphane translates to micromolar concentrations (0.1~10 μM) of the compound in various cells^8^, and doses required for HDAC inhibition and apoptosis induction appear to be much higher. However, SFN has been shown to accumulate in cells and reach to millimolar concentrations^8^. More trials will be needed to validate the usefulness of SFN either alone or in combination for the treatment of VS.

Taken together, the data presented here demonstrate the inhibitory effects of SFN on VS cells *in vitro* and *in vivo*. To the best of our knowledge, this is the first report showing that SFN exerts antitumor activity against VS through the inhibition of HDAC. SFN may therefore represent a potential chemotherapeutic agent for the treatment of VS.

## Methods

### Chemicals

DL-SFN (≥90% High Performance Liquid Chromatography, HPLC) was purchased from Sigma Aldrich (St. Louis, MO, USA). A stock solution of DL-SFN (5 mg/mL) was prepared with dimethyl sulfoxide (DMSO) and stored at −20 °C.

### Culture of primary human vestibular schwannoma cell and NF2-derived cell line

Using sterile technique, freshly harvested human VS tissue was rinsed in phosphate buffered saline (PBS) and dissected in culture medium consisting of Dulbecco’s Modified Eagle’s medium with Ham’s F12 mixture (DMEM/F12), 10% fetal bovine serum (FBS), 1% penicillin/streptomycin, and 1% GlutaMAX. VS tissue was then dissociated in hyaluronidase and collagenase (all Life Technologies, Carlsbad, CA, USA) overnight, and cultured for 2–4 weeks, as described previously^32^. Informed consent was obtained from all patients. The study protocols were approved by the Human Studies Committee of Massachusetts General Hospital and Massachusetts Eye and Ear Infirmary, and conducted in accordance with the Helsinki Declaration.

In addition to a primary human VS cell culture, two cell lines were used: 1) HEI-193 cells, an immortalized cell line derived from the vestibular schwannoma of a human NF2 patient, and 2) Nf2^−/−^ SC4 cells, a mouse Schwann cell line (both gifts from the House Ear Institute, Los Angeles, CA, USA). The cell lines were cultured in DMEM supplemented with 10% FBS and 1% penicillin/streptomycin.

### Cell proliferation assay: BrdU incorporation

To study proliferation of primary human VS cell lines, cells were incubated with 0 (control) or 10 μM SFN for 48 h, and then treated with 50 mg/mL bromodeoxyuridine (BrdU) (Invitrogen, Carlsbad, CA, USA) for 20 h. The cells were fixed, and the nuclear membranes were permeabilized with 1% Triton-X for 10 min, followed by 2N hydrochloric acid for 20 min. Primary antibodies against BrdU (AbD) (Serotec, Puchheim, Germany) and anti-rat IgG (Life Technologies) were used. Hoechst 33342 dye (hereafter abbreviated Hoechst, Invitrogen) were used to stain nuclei. BrdU- and Hoechst-stained nuclei were counted in 3–5 fields, and the ratio of BrdU-positive to Hoechst-positive nuclei was used to determine the proliferation rate *in vitro*.

### Cell viability assay

HEI-193 and SC4 cells were seeded in 96-well plates at 5 × 10^3^ and 3 × 10^3^ cells/well, respectively. After 24 h, the medium was replaced with serum-free medium and cells were incubated with various concentration of DL-SFN for 48 h. To quantify cell viability and metabolic activity, a calorimetric MTT [3-(4,5-dimethhylthiaoly)-2,5-diphenyltetrazolium bromide] (5 mg/mL) assay was used. Forty μg/mL of MTT solution (Sigma-Aldrich) in PBS was added to each well for 4 h at 37 °C. The culture medium containing MTT was then discarded. Visible formazan crystals were dissolved in 100 μL of DMSO, followed by shaking for 30 min at room temperature in the dark. The optical density (OD) was measured at 595 nm using a microplate reader. All experiments were repeated at least 3 times.

### Apoptosis assay: Annexin V/propidium iodide (PI) staining

Apoptosis was analyzed using an Annexin V assay kit (BD Biosciences Pharmingen, San Jose, CA, USA). Briefly, HEI-193 cells were seeded at 3 × 10^5^ cells/well in 6-well plates. After 24 h, the medium was replaced with serum-free medium and cells were incubated with 10–20 μM of DL-SFN for 48 h. Both floating and attached cells were then collected by trypsinization. After centrifugation at 1,300 rpm for 3 min, the cells were washed twice with PBS and incubated in 1 × binding buffer (10 m HEPES/NaOH (Ph 7.4), 140 mM NaCl, and 2.5 mM CaCl_2_) containing annexin V-fluorescein isothiocyanate (FITC) and propodium iodide (PI) at room temperature for 10 min in the dark. Stained cells were then analyzed using a FACScan flow cytometer (Becton, Franklin Lakes, NJ, USA) within 1 h. The data were analyzed using WinList 5.0 software (Verity Software House, Topsham, ME, USA).

### Apoptosis assay *in situ*: TUNEL assay

Apoptotic cells were detected *in situ* by utilizing the terminal deoxynucleotidyl trasferase-mediated dUTP nick end labeling (TUNEL) assay (Roche Molec Biochemic,Mannheim, Germany). HEI-193 cells were seeded on glass coverslips in 12-well plates at 8 × 10^4^ cells/well in DMEM supplemented with 5% FBS. After the monolayers achieved 70–80% confluence, cells were treated with 10–20 μM DL-SFN for 48 h. Cells on coverglass slides were then washed with PBS, fixed in cold 4% paraformaldehyde (Bio-Rad, Hercules, CA, USA) for 1 h, and treated 3% H_2_O_2_ in methanol for 10 min at room temperature to block endogenous peroxidase. After removal of the blocking solution, the cells were permeabilized in PBS-T (0.1% Triton X-100) for 5 min on ice, washed twice with PBS, incubated with TUNEL solution for 1 h at 37 °C, stained with 5 μg/mL Hoechst nuclear counterstain for 30 min at room temperature, and visualized by fluorescence microscopy.

### Cell cycle study

HEI-193 cells were used to study cell cycle arrest caused by SFN. HEI-193 cells were seeded in 6-well plates at 5 × 10^5^cells/well. After SFN application for 48 h, the floating cells and attached cells were collected by trypsinization, centrifuged at 1200 rpm for 5 min, fixed with ice-cold 70% ethanol, and stored at 4 °C for 1 h. Fixed cells were resuspended with PBS containing RNase A (100 ug/ml, Intron, Daejeon, Korea) for 30 min at 37 °C, and a propodium iodide (PI) solution (20 ug/ml, Invitrogen) was added. Stained cells were analyzed using a FACScan flow cytometer (Becton) and WinList 5.0 software(Verity Software House).

### HDAC assay

HDAC activity was quantified using the Histone Deacetylase Assay Kit, Fluorometric (Sigma-Aldrich) in accordance with the manufacturer’s protocol. Briefly, protein cell lysates were incubated with HDAC substrate (0.2 m M) in HDAC assay buffer at 30 °C for 30 min. Reactions were stopped by adding 10 μL developer solution, followed by incubation at room temperature for 10 min. Fluorescence was measured with excitation at 355 nm and emission at 460 nm using a fluorescence plate reader (Perkin Elmer, Boston, MA, USA).

### Western blot analysis

HEI-193 cells were used to interrogate activation of signaling molecules by Western blot. Specifically, HEI-193 cells were seeded in 10 cm dishes at a density of 1 × 10^6^ cells/well (Marienfeld, Germany). Cells were allowed to attach overnight in DMEM containing 10% FBS. To starve cells, the medium was replaced with medium containing 0.2% FBS. SFN was then added for 48 h. The cells were washed with PBS and collected by scraping in lysis buffer (Sigma-Aldrich) containing 7 × protease inhibitor cocktail and 10 × phosphatase inhibitor cocktail (Roche, Basel, Switzerland). Cell lysates were centrifuged at 13,000 rpm for 30 min at 4 °C, and protein concentration was determined using a lowry assay (Bio-Rad). Proteins (20 μg) were separated by SDS-polyacrylamide gel electrophoresis on a 6–15% gel, and transferred to PVDF membranes (EMD Millipore, Billerica, MA, USA). The membranes were blocked with 5% skim milk in TBS-T (0.1% Tween 20) at room temperature for 1 h, followed by incubation with primary antibodies against caspase-3 (Cell Signaling Technology, Inc., Danvers, MA, USA), caspase-9 (Santa Cruz Biotechnology, Inc., Santa Cruz, CA, USA), acetylated histone H3 (EMD Millipore), acetylated histone H4 (EMD Millipore), p-AKT (Santa Cruz), AKT, p-mTOR, mTOR, p-ERK, ERK (all from Cell Signaling Technology), and tubulin (EMD Millipore) overnight at 4 °C. Membranes were then washed in TBS-T, and incubated in horseradish peroxidase-conjugated goat anti-rabbit or goat anti-mouse antibodies, as appropriate, for 2 h at room temperature. Protein bands were detected by an ECL Plus Kit (Bio-Rad).

### *In vivo* tumorigenesis

Six-week-old female BALB/c nude mice (Nara Biotech Co., Seoul, Korea) were maintained under specific pathogen-free conditions and fed verified mouse chow. To cultivate tumors, SC4 Nf2^−/−^ Schwann cells were detached by trypsinization, collected in DMEM medium containing 10% FBS, and resuspended at a density of 3.4 × 10^5^ cells/mL in serum-free medium. A total volume of 0.3 mL (containing 1 × 10^5^ SC4 cells) was injected into the axillary region between the shoulder and chest wall. SFN (25 mg/kg) or an equal volume of DMSO (control group) was administered intraperitoneally 5 times per week for 6weeks. Each group consisted of eight mice. The tumor growth was evaluated using a caliper and tumor volume was estimated using the following formula: length (mm) × width (mm) × height (mm)/2. On the day after the final treatment, the animals were sacrificed, the solid tumors excised, and the weight of the tumors was measured. All animal work was performed in accordance with a protocol approved by the Institutional Animal Care and Use Committee of Korea Research Institute of Bioscience and Biotechnology.

### Statistical analysis

All data are presented as the mean ± standard error of mean (SEM). The independent t-test or one-way analysis of variance (ANOVA) with posthoc comparison using Bonferroni’s correction was employed for statistical analyses. The significance level was set at corrected p < 0.05. SPSS (version 14.0; SPSS, Inc, Chicago, Ill, USA) and R (version 3.1.3, The R Foundation for Statistical Computing, Vienna, Austria) were used to complete all analyses.

## Additional Information

**How to cite this article**: Kim, B. G. *et al*. Sulforaphane, a natural component of broccoli, inhibits vestibular schwannoma growth *in vitro* and *in vivo*. *Sci. Rep*. **6**, 36215; doi: 10.1038/srep36215 (2016).

**Publisher’s note:** Springer Nature remains neutral with regard to jurisdictional claims in published maps and institutional affiliations.
